# Editor’s Book Table

**Published:** 1870-10

**Authors:** 


### Editor's Book Table.

[NOTE.—All works reviewed in the columns of the CHICAGO MEDICAL JOURNAL may be found in the extensive stock of W. B. KEEN & COOKE, whose catalogue of Medical Books will be sent to any address upon request.]

*Medical Diagnosis, with Special Reference to Practical Medicine. A Guide to the Knowledge and Discrimination of Diseases.* By J. M. DACOSTA, M.D., etc. Illustrated with engravings on wood. Third Edition, revised and improved. Philadelphia: J. B. Lippincott & Co. 1870.

The success achieved by this treatise should be gratifying not only to the author himself, but to the profession. It is now an indispensable book to both students and practitioners. Clear, yet with no redundancy, it is a model of excellence in teaching. It is a good omen that books which elucidate facts about which there can be no question, yet whose knowledge is imperative upon the practitioner, are becoming popular, to the exclusion of pretentious catalogues of symptoms without reference to causation, followed by routine measures and methods of treatment, based almost wholly on *post hoc ergo propter hoc* reasoning, or the bald *ipse dixit* of some antediluvian, without the shadow of an idea of the true philosophy of medicines in his noddle. We gladly welcome such books, but from the "Pennyroyal-is-good-for-a-cold," or even the "Veratrum-Viride-is-good-for-fevers" style of literature, GOOD LORD DELIVER US!

What the profession wants, is to know, first, what the human body *is*, whether dead or living; then, *What is the matter?* when it is out of gear. Knowing these things, he is a blockhead who insults our intelligence, that undertakes to foist his pitiful specifics and mediæval mixtures upon us. That is sheer Homœopathy—no matter what name it goes by.

*The Physical Exploration of the Rectum: with an Appendix on the Ligations of Hæmorrhoidal Tumors.* By WILLIAM BODENHAMER, A.M., M.D. Illustrated by numerous drawings. New York: William Wood & Co., 61 Walker Street. 1870. Pp. 54.

A very useful, indeed, an admirable monograph, which each of our readers will find of practical use in every-day practice. We do not now call to mind any work which gives as complete an exposition of this important subject. In discussing the anatomy of the rectum, he denies the existence of valves from which important diagnostic results are noted. The best instruments and apparatus for physical exploration are described and illustrated, and he hopes for something valuable hereafter from the further development of "Splanchnoscopy by Translucency" as advocated by M. Milliot at the last International Congress at Paris. M. Milliot, it will be recollected, introduced into the stomach and rectum glass tubes of small calibre, containing two platinum wires connected with the electrodes of a battery. In this manner an intense internal illumination renders the visceral parietes translucent. Very particular directions and precautions are given with regard to the several points of manipulation.

The appendix discusses the ligation of hæmorrhoidal tumors. The author advocates the use, almost exclusively, of the silk ligature, well waxed and with as little twist as possible in it. But for details we must refer our readers to the monograph itself, which we advise them to purchase.

*The American Dispensatory.* By JOHN KING, M.D., Professor of Obstetrics and Diseases of Women and Children, in the Eclectic Medical Institute of Cincinnati, etc., etc. Eighth Edition, completely revised and largely rewritten. One vol., royal octavo, 1440 pages, sheep, price $10.00. Cincinnati: Wilstach, Baldwin & Co., Publishers, 141 and 143 Race St.

Our Eclectic and Homœopathic friends are great believers in medicines, of which this ponderous volume is a striking evidence. A glance at its index almost overwhelms one with astonishment that anybody should be foolish enough to die, or even be sick, whilst so many full grown remedies for every imaginable ailment are furnished to his organs of deglutition or absorption. The disciples of Hahnemann not only bring in a swarm of new medicines, but, by their mysterious "provings," show that each of them is possessed of a hundredfold more varieties of symptomatic effects, than the proverbial nine-and-thirty stinks of Cologne. This is a somewhat singular fact when taken in connection with the notable truth that, contemporaneously with the advance of scientific medicine, the number and importance of so-called medicines are becoming wonderfully contracted. It is safe to say that educated medical men trust very little in drugs, but seek to control, for the preservation and restoration of health, those larger influences which our fathers so strangely termed the "Non-naturals." In our own view, the man of multitudinous drugs and complicated formulæ, is dangerous to the physical well-being and safety of the community. In a city like this, one can follow such men through their rounds by the crape on the handles of the door bells. It is proper to say that the science of medicine in this age is being advanced, not so much by the discovery of new drugs, or new effects from those previously in use, as by better acquaintance with physiology and other natural sciences. It is in rare instances only, that even the most popular so-called medicines have achieved their position save by modes of reasoning which, in any other department of human knowledge, would be counted simply absurd. Rational men see this, and some are governed in practice by it; nevertheless very many still believe in Chinese gongs and Indian howlings instead of Nature's laws.

The volume before us must rejoice the heart of your true believer in drugs. It is stuffed, from board to board, with accounts of health-bearing herbs and roots, minerals, chemical combinations, "pharmaceutical compounds, alkalies, resinoids, oleo-resins, etc., and likewise all those poisonous mineral agents so strongly objected to by the new school physicians, thus forming a volume full and complete in itself." It is a work evincing abundant industry on the part of its author, and we commend it to those of our subscribers who want us to chronicle the wonders of mint, cummin and anise, at risk of neglecting the weightier matters of the law. We commend it to those of our compeers in the city who are so hurried by business, and yet so desirous of information, that they read in their carriages while passing through the most crowded streets; their attendant Jehus, meanwhile, borrowing from their association an impressive dignity wondrous to behold. Dear brethren, this book will enable you by its size, at least, to hide your blushes from the gaze of your multitudinous admirers.

Dr. King must have the credit of arranging the voluminous material at his command in excellent order, and gives perspicuously, and, so far as our examination permits us to judge, with remarkable correctness, the physical history of the multitudinous "Botanical" Materia Medica. Very many of the so-called eclectic medicines are now in daily use—employed by homœopathists and physicians regularly, and in this great volume the seeker can find the claims of each and all.

